# Diagnosis of Aortic Dissection in Emergency Department Patients is Rare

**DOI:** 10.5811/westjem.2015.6.25752

**Published:** 2015-10-20

**Authors:** Scott M. Alter, Barnet Eskin, John R. Allegra

**Affiliations:** * Carolinas Medical Center, Department of Emergency Medicine, Charlotte, North Carolina; † Morristown Medical Center, Department of Emergency Medicine, Morristown, New Jersey

## Abstract

**Introduction:**

Aortic dissection is a rare event. While the most frequent symptom is chest pain, that is a common emergency department (ED) chief complaint and other diseases causing chest pain occur much more often. Furthermore, 20% of dissections are without chest pain and 6% are painless. For these reasons, diagnosing dissections may be challenging. Our goal was to determine the number of total ED and atraumatic chest pain patients for every aortic dissection diagnosed by emergency physicians.

**Methods:**

Design: Retrospective cohort. Setting: 33 suburban and urban New York and New Jersey EDs with annual visits between 8,000 and 80,000. Participants: Consecutive patients seen by emergency physicians from 1-1-1996 through 12-31-2010. Observations: We identified aortic dissection and atraumatic chest pain patients using the International Classification of Diseases 9^th^ Revision and Clinical Modification codes. We then calculated the number of total ED and atraumatic chest pain patients for every aortic dissection, along with 95% confidence intervals (CIs).

**Results:**

From a database of 9.5 million ED visits, we identified 782 aortic dissections or one for every 12,200 (95% CI [11,400–13,100]) visits. The mean age of dissection patients was 66±16 years and 38% were female. There were 763,000 (8%) with atraumatic chest pain diagnoses. Thus, there is one dissection for every 980 (95% CI [910–1,050]) atraumatic chest pain patients.

**Conclusion:**

The diagnosis of aortic dissections by emergency physicians is rare and challenging. An emergency physician seeing 3,000 to 4,000 patients a year would diagnose an aortic dissection approximately every three to four years.

## INTRODUCTION

Emergency physicians (EPs) strive never to miss the diagnosis of aortic dissection because this can be devastating to the patient and also stressful to the physician.^1^ However, aortic dissection is a rare disease and identifying dissection may be challenging. Its symptoms, most commonly chest pain, often overlap those of conditions much more commonly found in the emergency department (ED), including acute coronary syndrome and pulmonary embolus.^2^ It is easy to order and perform the diagnostic test most commonly used to diagnose dissection, computerized tomography (CT) angiography of the chest. However, ordering a CT for everyone for whom dissection is a consideration, even those with a remote possibility, may not be the best strategy. Patients may suffer adverse effects from a CT angiogram, such as acute renal failure or allergic reactions, and all will have radiation exposure with consequent cancer risks.^3^ Also, CTs are costly, lengthen patient stays, and inconvenience patients.

The rarity of dissection makes it inevitable that EPs will miss or delay diagnosing some. Our goal was to estimate the magnitude of this problem by studying how seldom dissections are diagnosed in the ED. We did not find previous studies addressing this in the literature. An estimate of the incidence may be useful for clinicians as they weigh the risks and benefits of ordering CTs**,** and for physicians currently involved in litigation regarding failure or delay in diagnosing aortic dissection.

## METHODS

### Design and setting

We conducted a retrospective cohort study of patients at 33 suburban and urban New York and New Jersey EDs, with annual visits between 8,000 and 80,000. Our institutional review board approved this study.

### Selection of participants

We included consecutive patients seen by EPs from January 1, 1996 through December 31, 2010.

### Methods and measurements

EPs documented diagnoses in their charts at the time of patient encounter. Trained coders in the billing department then assigned International Classification of Diseases 9^th^ Revision and Clinical Modification (ICD-9) codes to the chart. We identified aortic dissection visits from ICD-9 codes (441.00, 441.01, 441.02, and 441.03), and then exported visit information to Excel (Microsoft Corporation, Redmond WA) for analysis. *A priori*, we generated an expansive list of ICD-9 codes for atraumatic chest pain. Since the pain in aortic dissection patients can have varying quality, location, and intensity, we included all diagnoses with presenting symptoms that aortic dissection patients could have.

### Analysis

We calculated the number of total ED and atraumatic chest pain patients for every aortic dissection, along with 95% confidence intervals (CIs).

## RESULTS

The ED database contained a total of 9,533,827 patient visits. Of these, there were 782 aortic dissections, or one for every 12,200 visits (95% CI [11,400–13,100]). The mean age of aortic dissection patients was 66±16 years and 38% were female.

Seventy-three ICD-9 codes were determined to meet criteria for presentation of symptoms potentially having a diagnosis of aortic dissection. These included diseases such as cholecystitis, cardiac tamponade, acute myocardial infarction, and heartburn, as well as unspecified chest pain, epigastric pain, etc. (A full list of ICD-9 codes used is available in the Appendix.) Of the total ED visits there were an estimated 763,000 (8%) with atraumatic chest pain diagnoses. There was one dissection for every 980 atraumatic chest pain patients (95% CI [910–1,050]).

## DISCUSSION

Aortic dissection is one of the most important diseases not to miss, yet its diagnosis in the ED is very rare. We found one aortic dissection for every 12,200 ED patients and one for every 980 patients with atraumatic chest pain. An EP seeing 3,000 to 4,000 patients a year^4^ would diagnose an aortic dissection approximately once every three to four years.

In addition, aortic dissections often present with a wide range of symptoms. One study found only 71% of patients with type A dissections had anterior chest pain and 6% had no pain at all.^2^ Furthermore, aortic dissection may present with symptoms, such as heart failure, neurologic deficits, syncope, or vascular insufficiency, which are found more commonly in other diseases.^5^ Kurabayashi et al. found aortic dissections were misdiagnosed in 16% of cases presenting to the ED.^6^ This is likely an underestimate as patients with dissection, particularly those who die, may never receive the correct diagnosis.

History and physical examination alone is unreliable in diagnosing aortic dissections as physicians correctly suspect aortic dissection after the initial clinical evaluation in only 65% of patients.^7^ While sudden onset of severe pain with elevated blood pressure and pulse deficits suggest dissection, absence of these findings does not exclude it.^8^ Consequently, researchers have devised scoring systems to risk stratify patients; however, none have performed well or achieved widespread use.^9^–^10^ D-dimer may be suitable as a “rule-out” tool with a useful negative likelihood ratio, though the positive likelihood ratio is not helpful.^11^ Chest radiograph can be used as a screening tool, as finding multiple abnormalities has a sensitivity of 90% in detecting aortic dissection.^8^ However individual findings, such as abnormal aortic contour and widened mediastinum, have sensitivities from 9% to 71%.^8^ Another study found chest radiograph sensitivity and specificity for aortic dissection of only 67% and 70%, respectively.^12^

None of the approaches above is sufficient for diagnosing aortic dissection, and performing chest CT imaging on every patient may not be the best strategy. Unfortunately, failure or delay in diagnosis may lead to significant morbidity and mortality. In addition, this may lead to litigation: in a series of aortic dissection lawsuits, 58% were related to failure or delay in diagnosis.^13^ Patients and their families blame physicians for poor outcomes and then seek high monetary compensation.^14^ This is distressing to the practitioner, and fear of litigation may lead to the diversion of resources in a futile effort to achieve diagnostic perfection.^1^ Like other relatively rare diseases such as bacterial meningitis and subarachnoid hemorrhage, delay in treating or failure to diagnose aortic dissections carries significant morbidity, mortality, and litigation implications. Nevertheless, if we miss the diagnosis, the patient may die – an outcome that no physician wants to see. Unfortunately, the argument that “it is a rare diagnosis” is not likely to be an effective defense in court.

## LIMITATIONS

Our study was limited by its retrospective nature. We identified aortic dissections from a database using ICD-9 codes based on EP diagnoses. The better way would be to define prospectively which patients to include as having an aortic dissection. However, given the relative rarity of the diagnosis, a prospective study would need to enroll a very large number of patients. For example, accumulating 100 dissection patients would require a total ED patient volume of 100 times 12,200, which is about 1.2 million patients – an overwhelming task. Additionally, using ICD-9 codes may have led to over or under counting; however, we do not believe that this would greatly change our results. In addition, many diagnoses of dissection are not made in the ED, but after admission, for example during interventional angiography performed for suspected acute coronary syndrome.

In our study, we identified atraumatic chest pain patients using an expansive list of diagnoses because the characteristics of chest pain associated with aortic dissections are varied. Using a narrower list of diagnoses would have identified 5% to 6% of all ED patients as having chest pain. This would have led to an estimate of one aortic dissection for every 600 to 700 patients presenting with chest pain.

## CONCLUSION

We found aortic dissections to be rare, diagnosed approximately once for every 12,200 ED patients and once for every 980 atraumatic chest pain patients. Although ordering CTs in low-probability patients may not be the best strategy, missing the diagnosis can have devastating consequences for the few patients that actually have a dissection. These findings may be useful for clinicians as they weigh the risks and benefits of ordering CTs, and also for physicians currently involved in litigation regarding failure or delay in diagnosing aortic dissection.
