# Supplementary material for: Diagnosis of Aortic Dissection in Emergency Department Patients is Rare
Source: West J Emerg Med. 2015 Oct 20;16(5):629–31. doi: 10.5811/westjem.2015.6.25752 (PMC4644027; doi:10.5811/westjem.2015.6.25752)
Supplement: Supplementary file 1 [file wjem-16-629-s001.pdf]

Appendix: ICD-9 codes of diagnoses potentially presenting with atraumatic chest pain.

ICD-9 code	Diagnosis
396.9	Mitral and aortic valve diseases unspecified
398.90	Rheumatic heart disease unspecified
402.91	Unspecified hypertensive heart disease with heart failure
410.00	Acute myocardial infarction of anterolateral wall episode of care unspecified
410.10	Acute myocardial infarction of other anterior wall episode of care unspecified
410.11	Acute myocardial infarction of other anterior wall initial episode of care
410.12	Acute myocardial infarction of other anterior wall subsequent episode of care
410.20	Acute myocardial infarction of inferolateral wall episode of care unspecified
410.21	Acute myocardial infarction of inferolateral wall initial episode of care
410.31	Acute myocardial infarction of inferoposterior wall initial episode of care
410.40	Acute myocardial infarction of other inferior wall episode of care unspecified
410.41	Acute myocardial infarction of other inferior wall initial episode of care
410.50	Acute myocardial infarction of other lateral wall episode of care unspecified
410.51	Acute myocardial infarction of other lateral wall initial episode of care
410.60	True posterior wall infarction episode of care unspecified
410.61	True posterior wall infarction initial episode of care
410.70	Subendocardial infarction episode of care unspecified
410.71	Subendocardial infarction initial episode of care
410.72	Subendocardial infarction subsequent episode of care
410.81	Acute myocardial infarction of other specified sites initial episode of care
410.90	Acute myocardial infarction of unspecified site episode of care unspecified
410.91	Acute myocardial infarction of unspecified site initial episode of care
410.92	Acute myocardial infarction of unspecified site subsequent episode of care
411.0	Postmyocardial infarction syndrome
411.81	Acute coronary occlusion without myocardial infarction
414.00	Coronary atherosclerosis of unspecified type of vessel native or graft
414.10	Aneurysm of heart (wall)
414.9	Chronic ischemic heart disease unspecified
415.11	Iatrogenic pulmonary embolism and infarction
415.12	Septic pulmonary embolism
415.19	Other pulmonary embolism and infarction
416.9	Chronic pulmonary heart disease unspecified
417.1	Aneurysm of pulmonary artery
417.8	Other specified diseases of pulmonary circulation
420.0	Acute pericarditis in diseases classified elsewhere
420.90	Acute pericarditis unspecified
420.91	Acute idiopathic pericarditis
421.9	Acute endocarditis unspecified
422.90	Acute myocarditis unspecified

422.92	Septic myocarditis
423.1	Adhesive pericarditis
423.2	Constrictive pericarditis
423.3	Cardiac tamponade
423.8	Other specified diseases of pericardium
428.1	Left heart failure
428.9	Heart failure unspecified
429.0	Myocarditis unspecified
429.1	Myocardial degeneration
429.2	Cardiovascular disease unspecified
429.89	Other ill-defined heart diseases
440.0	Atherosclerosis of aorta
440.9	Generalized and unspecified atherosclerosis
441.00	Dissection of aorta aneurysm unspecified site
441.01	Dissection of aorta thoracic
441.03	Dissection of aorta thoracoabdominal
441.1	Thoracic aneurysm ruptured
441.2	Thoracic aneurysm without rupture
441.5	Aortic aneurysm of unspecified site ruptured
441.9	Aortic aneurysm of unspecified site without rupture
444.1	Embolism and thrombosis of thoracic aorta
519.3	Other diseases of mediastinum not elsewhere classified
575.0	Acute cholecystitis
745.4	Ventricular septal defect
746.89	Other specified congenital anomalies of heart
746.9	Unspecified congenital anomaly of heart
747.22	Congenital atresia and stenosis of aorta
785.2	Undiagnosed cardiac murmurs
786.50	Unspecified chest pain
786.51	Precordial pain
786.52	Painful respiration
786.59	Other chest pain
787.1	Heartburn
995.59	Other child abuse and neglect

2
